# Effect before Cause: Supramodal Recalibration of Sensorimotor Timing

**DOI:** 10.1371/journal.pone.0007681

**Published:** 2009-11-05

**Authors:** James Heron, James V. M. Hanson, David Whitaker

**Affiliations:** Bradford School of Optometry and Vision Science, University of Bradford, Bradford, United Kingdom; Baylor College of Medicine, United States of America

## Abstract

**Background:**

Our motor actions normally generate sensory events, but how do we know which events were self generated and which have external causes? Here we use temporal adaptation to investigate the processing stage and generality of our sensorimotor timing estimates.

**Methodology/Principal Findings:**

Adaptation to artificially-induced delays between action and event can produce a startling percept—upon removal of the delay it feels as if the sensory event precedes its causative action. This temporal recalibration of action and event occurs in a quantitatively similar manner across the sensory modalities. Critically, it is robust to the replacement of one sense during the adaptation phase with another sense during the test judgment.

**Conclusions/Significance:**

Our findings suggest a high-level, supramodal recalibration mechanism. The effects are well described by a simple model which attempts to preserve the expected synchrony between action and event, but only when causality indicates it is reasonable to do so. We further demonstrate that this model successfully characterises related adaptation data from outside the sensorimotor domain.

## Introduction

Self-generated sensory stimuli will typically share a common temporal register–their physical onset times will be closely correlated with the moment in time when the causative motor action is completed. For example, if we imagine the act of clicking our fingers together, completing the motor action generates instantaneous visual, tactile, and auditory sensory information. Recently, Stetson et al. [1] demonstrated that the perceived timing of the visuo-motor component of this action-event ensemble can be markedly influenced by recent experience. Adaptation to a fixed delay between a button press and ensuing visual flash induces non-veridical perception of subsequent, physically simultaneous button press-flash pairings: their reported temporal order is reversed–‘I saw the flash before I pressed the button!’ - providing empirical quantification of earlier, qualitative reports [2]. More recent reports suggest that such effects can persist for at least 40 seconds in the absence of updated visual feedback [3]. This type of temporal recalibration is reminiscent of the nervous system's response to spatial misalignment between seen and felt location during prism adaptation experiments (e.g. [4]). It also has parallels with purely *sensory* effects observed following adaptation to audiovisual asynchrony [5]–[7]. Recently, Hanson et al. [8] speculated that a single ‘supramodal’ mechanism may be responsible for the recalibration of perceived time across sensory pairings. Other behavioural studies provide support for this concept. For example, despite absolute differences between observers, within-observer differences in temporal sensitivity are well correlated between perceptual and motor timing tasks [9]–[11]. Moreover, perceptual learning effects observed during interval timing tasks are highly specific to the trained base interval yet readily transfer between visual hemispheres [12], sensory modalities [13], and perceptual to motor tasks [14], [15]. These findings imply that time itself–rather than the nature of the sensory or motor information by which it is defined–may be the critical perceptual parameter. Returning to the example of Stetson et al.'s [1] visuo-motor effects, a single, late-stage timing mechanism might be expected to recalibrate all the self generated sensory consequences of motor actions in a similar manner [16].

Despite this, a host of recent studies have demonstrated that visual stimuli such as that employed by Stetson *et al.* are subject to perceptual distortions with seemingly low-level neural loci. For example, simply reducing the visibility or increasing spatial frequency of visual stimuli induces dramatic compressions in their perceived duration [17]. Similar effects have been observed following adaptation to drifting gratings [18], [19], flickering patches [19] or simply executing a saccadic eye movement [20]. Significantly, these adaptation effects have been shown to be specific to the visual modality [19] and the region of visual space occupied by the adapting stimulus [18], [19]. In the auditory domain, psychophysical interval timing data is successfully predicted by a model whose temporal estimates are derived from the spatiotemporal distribution of neural activity (or ‘state dependent networks’) which could be performed at a range of neural processing scales [21]. This model is supported by a transcranial magnetic stimulation (TMS) study showing that TMS to visual cortical areas degrades visual (but not auditory) temporal sensitivity [22]. Taken together, these findings are difficult to reconcile with centralised clock models and point toward early, peripheral timing mechanisms that are selective for modality and low-level stimulus features.

Given this dichotomy in the literature, it is conceivable that the nervous system could employ (i) a single, central, supramodal mechanism charged with encoding all visuo-motor, auditory-motor and tactile-motor temporal information, *or* (ii) each sensorimotor domain could employ its own individual, peripheral mechanism. Here we examine this issue by investigating the interaction between sensorimotor timing, causality and the role of recent experience across the sensory modalities.

## Results

Observers adapted to a fixed delay (either 50, 100, 200, 400 or 800 ms) between the completion of their motor action (a mousepress) and either visual (an LED ‘flash’), auditory (a white noise ‘click’) or tactile (a ‘tap’ delivered to the opposite index finger) feedback. Observers were instructed to press the mouse button at intervals of their own choosing so as to ensure their motor actions were entirely voluntary in nature [23], [24]. Following adaptation, observers were presented with a range of ‘test’ stimuli in which their ‘test’ motor action (mousepress) was followed by sensory feedback (from within the same modality as the adapting stimuli) with a variable delay (25–125 ms). Observers made binary forced choice temporal order judgments (TOJs) as to ‘which came first, my mousepress or the flash/click/tap?’ (see ‘Materials and Methods’ for details).

**Figure 1:**
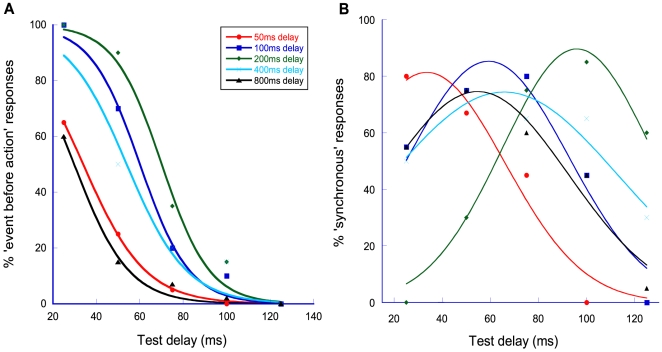
Temporal order and synchronous/asynchronous judgments after adaptation to sensorimotor delay. Raw data for representative, naïve observer KJW. (**A**) A sample of the psychometric functions generated via temporal order judgments: ‘which came first, action or event?’. This sample shows unimodal data from the audio-motor section of the adaptation experiment. The percentage of ‘event before action’ trials (i.e. where observers report an illusory reversal of temporal order) is plotted as a function of test delay (the physical asynchrony between their action (a mousepress) and a sensory event (an auditory ‘click’ in this example)). The different coloured functions represent different sensorimotor adaptation delays (see Figure key for details). (**B**) Data for the same observer for a control experiment. The only difference between the data shown in A&B is the nature of the judgment type. In this data set, observers were presented with the same stimuli (‘mousepress-click’) but made synchronous/asynchronous (as opposed to temporal order) judgements (see main text for details). The same Figure key applies to both panels.

Figure 1A provides an example of the resultant psychometric functions for the condition where observers adapted to delayed audio-motor feedback following their motor actions. The percentage of ‘event before action’ responses is plotted against test delay. Taking the example of the ‘200 ms’ adaptation data (green curve - diamonds), it can be seen that at small test delays (e.g. 25 ms) observers perceive an illusory reversal of temporal order: despite physical delays between their motor actions and subsequent sensory events, they report the action to follow the event. As test delay increases, the perception of temporal order becomes more veridical, with the percentage of ‘event before action’ responses falling toward zero. The fact that these functions are laterally displaced (relative to one another) shows that this effect is dependent on the magnitude of the adapting delay. Specifically, functions pertaining to the 50 ms condition (red curve - circles) through to the 200 ms condition show a progressive rightward shift in their mid-points. Larger adapting delays result in a progressive reduction of illusory responses.

**Figure 2:**
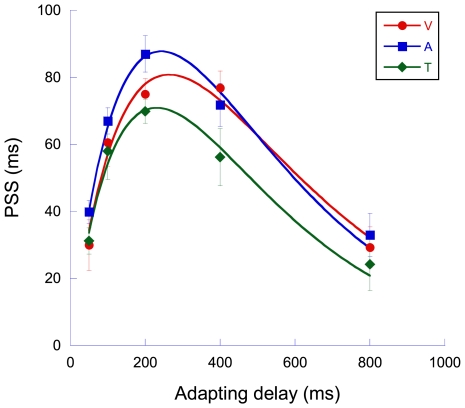
Adaption shifts the point of subjective sensorimotor simultaneity across the sensorimotor pairings. Temporal recalibration of motor action and a sensory event in the visual (red circles), auditory (blue squares) and tactile (green diamonds) sensorimotor pairings. Data points represent the physical sensorimotor asynchrony necessary to produce perceptual synchrony (PSS). Positive values signify a temporal lead of action over event. The data are fitted with a model with two free parameters (see main text for details). Error bars represent one standard error of the mean (variance between observers) either side of the parameter values (n = 5).

The midpoints of these functions represent the point of subjective simultaneity (the physical temporal offset corresponding to perceptual sensorimotor simultaneity or ‘PSS’). Their dependence on adapting delay is illustrated in Figure 2 where the PSS values have been extracted, averaged across observers and plotted as a function of adapting delay. Inspection of Figure 2 shows that adaptation to small delays between motor actions and their associated visual (red circles), auditory (blue squares) or tactile (green diamonds) sensory feedback produces vigorous perceptual recalibration of perceived sensorimotor time - observers require an increasing *physical* delay between their mousepress and subsequent sensory feedback in order to perceive *perceptual* sensorimotor simultaneity. The magnitude of this recalibration appears to form a fixed proportion of the adapting delay when the delay is below ∼200 ms [5], beyond which a reduction in effect size is observed. The significant effect of delay was confirmed by a repeated measures analysis of variance (F_4,16_ = 37.1, p<0.001). The difference between PSS values for each of the three senses just reached significance (F_2,8_ = 5.57, p = 0.030) although there was no significant interaction between delay and effect size (F_8,32_ = 1.30, p>0.05).

Some consider that TOJs may be susceptible to response bias or ‘criterion-based’ artefacts that can shift PSS via strategic factors that are likely to be of a cognitive, rather than perceptual nature [25], [26]. It could be argued that observers–subconsciously or otherwise–adopted a strategy of distributing their TOJ responses around some criterion other than their perceived arrival times. This could have the effect of shifting the functions midpoint in the opposite direction to the response bias. It has been suggested that a ‘synchronous/asynchronous’ judgment type is less susceptible to this issue [26], [27]. We therefore conducted a control experiment where the audio-motor paradigm was revisited. The stimuli and procedures were identical to those used in the previous experiment with the exception that observers were now asked to make forced choice judgments about whether their actions and subsequent sensory feedback were synchronous or asynchronous. The data are shown in Figure 1B alongside the psychometric functions for the same representative, naïve observer. Fitting these data with Gaussian functions (see ‘Materials and Methods’ for details) allows visualisation of how PSS (the function's mean or ‘peak’ value) varies with adapting delay. If our effects were peculiar to TOJs we would expect the Gaussians to be superimposed on top of one another with means centered around zero. This is clearly not the case. Comparison with Figure 1A reveals that, despite some small differences (e.g. in the 200 ms delay condition, the PSS shift is slightly larger for synchronous/asynchronous judgements than for TOJs), the overall pattern of results is strikingly similar: a progressive rightward shift in the mean's lateral position from the 50–200 ms conditions with larger delays reversing the trend. This finding suggests that our effects are relatively robust and cannot be ascribed to idiosyncrasies in observer judgment type.

It should be stressed that our illusory effects in both the temporal order and synchronous/asynchronous experiments were quantifiable despite the absence of trials where where sensory events physically preceded motor actions. Small to medium sized delays resulted in large numbers of ‘event before action’ responses across naïve and non-naïve observers. Indeed, naive observers were extremely surprised to learn that no trials actually included a sensory stimulus prior to their motor response. This highlights the robustness of the effect which has now been demonstrated using a number of different experimental paradigms [1]–[3].

Returning to the data shown in Figure 2, it seems that combinations of factors are contributing to the magnitude of our adaptation effects. One is the lifelong experience that motor actions and sensory events tend to be closely associated in time. When presented with a temporal asynchrony between action and event (both of which possess temporal uncertainty), a likely inference is that this asynchrony is the result of neural error indicating the need for re-calibration. This could either be achieved by realigning the noisy sensory estimate of delay toward a ‘synchrony prior’ [28], changing the synchrony prior in the light of adaptation [29], [30] or manipulating the Bayesian combination of both with an additional noisy estimate based on current context [31]. This type of sensory realignment appears in many different forms, but Helson's Adaptation Level Theory [32] underlies all of them. This theory proposes that the current, adapted state provides a sensory standard against which new stimuli are perceived. The result is that, in trials where the adapting stimulus is suddenly removed (‘catch’ trials), significant ‘rebound’ judgment errors result. Motor adaptation is commonly studied in this way, with the general finding that the extent of adaptation is proportional to the motor disturbance introduced. In the context of our adaptation effects, this would suggest that temporal recalibration should increase linearly with adapting delay. This is not the case (Figure 2), and brings us on to the second factor determining our effects.

In the motor domain, it has recently become evident that the relevance or ‘credibility’ of disturbing forces affecting motor actions are taken into consideration before making adaptive changes [33], [34]. Similarly, in the sensorimotor domain, human observers typically show a rapid fall-off in their tendency to attribute sensory feedback as being a consequence of their motor actions as the temporal discrepancy between the two is increased [35]–[38].

The concept of causality appears to be critical [39], with Haggard et al [40] finding that the perceived times of motor actions and events are indeed attracted towards each other in time, but that this effect dissipates rapidly with the physical delay between the two. Inspection of Haggard's data reveals that the rate of decline is well described by an exponential function [40]. We therefore suggest that our adaptation effects decrease at long delays because observers no longer consider the sensory event to be a direct consequence of their action.

On this basis, we model our data as a combination of two factors–a linear increase in temporal recalibration which is proportional to the delay, combined with an exponential reduction in the tendency to attribute action and event as being associated. The data of Figure 2 are therefore fitted with the function
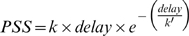
where k is a constant of proportionality and k' determines the rate of exponential decay.

This provides an excellent fit to the data sets for each sense. Parameter values along with their errors and goodness-of-fit are given in Table 1. The proximity to unity of the parameter k, for all three senses, indicates that temporal recalibration is virtually complete at small delays. Observers fully adapt to the delay and recalibrate their sense of synchrony accordingly.

**Table 1:** 

Sense	k	k' (msec)	R^2^
**Vision**	0.831±0.053	264±14	0.971
**Audition**	0.984±0.042	242±8	0.983
**Touch**	0.836±0.048	231±11	0.973

Whilst the visuo-motor data corroborates the recent data of Stetson et al. [1], the auditory and tactile data form the first demonstration of adaptive temporal realignment of audio-motor and tactile-motor perception. The most striking feature of the data shown in Figure 2 is the similarity in the pattern of results across the three sensorimotor domains. A potential explanation for equivalence between the modalities is a single perceptual mechanism that recalibrates *all* the sensory consequences of a given motor action. If the recalibration mechanism is manifest at a sufficiently late-stage of temporal processing (e.g., beyond modality-specific cortical areas) the recalibration effects shown in Figures 1 & 2 should survive the replacement of the sensory component between adapt and test phases. To address this question we performed a further, similar experiment where observers adapted to a fixed 200 ms delay (where the adaptation effects shown in Figure 2 appear maximal) in one sensorimotor pairing but their post-adaptation sensorimotor TOJs were tested with a different sensorimotor pairing (e.g., adapt ‘mousepress-flash’, test ‘mousepress-click’) (see ‘Materials and Methods’ for details).

**Figure 3:**
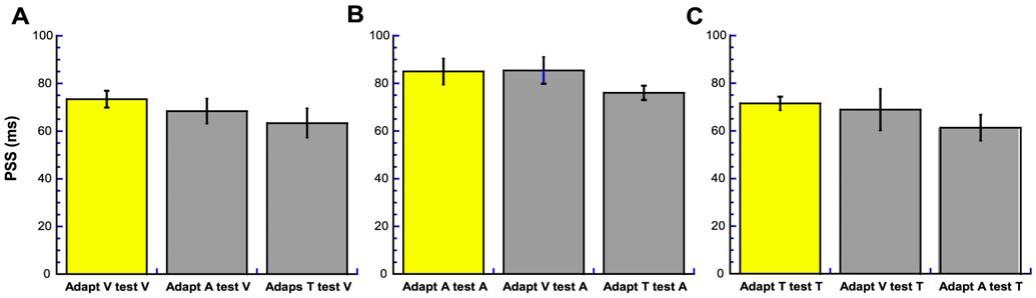
Temporal recalibration transfers to un-adapted sensorimotor pairings. Average PSS values (ms) from **(A)** visuo-motor (V), **(B)** auditory-motor (A) and **(c)** tactile-motor (T) sensorimotor temporal order judgments following adaptation to a 200 ms delay between action and event. Positive values signify a temporal lead of action over event. Yellow bars represent within-modality data taken from the 200 ms condition in Figure 2. Grey bars represent the crossmodal conditions for the same delay. Error bars represent one standard error of the mean either side of the parameter values (n = 7).

Figure 3 shows the average PSS values extracted from the resultant crossmodal psychometric functions (Fig. 3 - grey bars), alongside the within-modality 200 ms values from Figure 2 (Fig. 3 - yellow bars). It is clear that the recalibration effects are unaffected by the changeover of the sensory component between adaptation and test phases. The similarity between the effect size across conditions is confirmed by two-way repeated measures ANOVA, which revealed that the effect of test modality on PSS was significant (F_2, 12_ = 7.41, p = 0.008), but that the effect of the adapting modality was not significant (F_2, 12_ = 1.61, p>0.05). The former reflects the fact that the magnitude of the effect was slightly, but consistently, higher for the ‘test A’ condition (Figure 3B). Critically, however, there was no significant interaction between these two factors (F_4, 24_ = 2.02, p>0.05). In other words, the magnitude of the effect for a given test modality does not depend on the adapting modality. Thus, adaptation to delayed sensory consequences recalibrates *all* the sensory consequences of the motor action employed in the current study.

**Figure 4:**
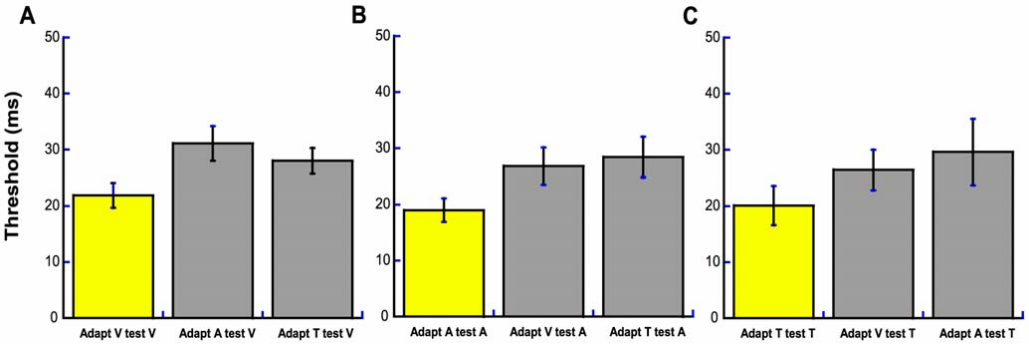
Sensitivity to changes in relative sensorimotor time. Average threshold values (ms) from (**A**) visuo-motor, (**B**) audio-motor and (**C**) tactile-motor sensorimotor temporal order judgments following adaptation to a 200 ms delay between action and event. In each plot, yellow bars represent within-modality data taken from the 200 ms condition in Figure 2. Grey bars represent crossmodal data (e.g., adapt visuo-motor, test audio-motor). Error bars represent one standard error of the mean either side of the parameter values (n = 7).

Thus far, we have considered changes in perceived temporal alignment (PSS), as opposed to the sensitivity to changes in relative temporal position. In the first, within-modality experiment, sensorimotor temporal order thresholds were not dependent on test stimulus modality (F_2, 8_ = 1.23, p>0.05). In the crossmodal ‘200 ms’ condition, threshold values were not dependent on test modality or adapting modality *per se* (F_2, 12_ = 0.712, p>0.05), but the interaction between adapt and test modalities was significant (F_4, 24_ = 9.815, p = 0.0001). Figure 4 shows this arises from the notable cost to performance when the modality of the sensory event changes between adapt and test phases. This is in keeping with studies showing that temporal performance is compromised by rapid switching of attention between the sensory modalities (e.g., [12], [41]).

## Discussion

In the current study we set out to investigate how recent experience influences the nervous system's estimates of sensorimotor timing across the sensory modalities. The data from the first experiment clearly show that the visual, auditory and tactile sensory consequences of our motor actions are all subject to marked temporal recalibration when presented with consistent temporal delays. The results of the final, crossmodal experiment reveal that this recalibration transfers to sensorimotor pairings other than those included in the adaptation phase. Our data are well described by a model in which an observer's prior experience leads them to impose perceptual synchrony between motor actions and their sensory consequences. One intuitively appealing feature of this model is that it balances the costs and benefits of recalibrating perception in response to an altered physical environment. Small sensorimotor temporal delays are treated as improbable, and the perceived sensorimotor timing of *all* potential sensory feedback is almost completely realigned. Increasing delays between actions and afferent sensory inputs are classified as exponentially more likely to have arisen from independent causes (i.e., external agencies), thus minimising the risk of erroneous adaptation.

Elements of our model have parallels with two concepts from the causality literature. Firstly, the notion of an ‘internal comparison process’ first postulated by Helmholtz (for a recent review see [42]). Here, observers compute the difference between the predicted and perceived afferent sensory feedback following completion of their motor commands (e.g., a saccade [43] or contact between fingers [44]). The output of this ‘comparator model’ gives a metric of causality, and it could be argued that our observers use such an output to recalibrate sensorimotor time. For example, Bays et al [35] discuss a general principal of how prediction is employed in a variety of situations such as tactile force perception. Tactile sensation is typically attenuated when it is associated with self-generated actions [35], [45]. Interestingly, this attenuation can occur without the full completion of the action, presumably because the nervous system makes prior assumptions about the sensory consequences [44]. Secondly, an assumption of synchrony also has implications for the nature of ‘intentional binding’ effects described by Haggard and colleagues, in which observers consistently underestimate the temporal interval between voluntary actions and their sensory consequences (e.g., [40], [46]). This underestimation can be thought of as either (i) a local compression of the temporal interval between action and event or, (ii) a temporal realignment (i.e. recalibration) of the signals binding the interval [1]. Our adaptation data favour the latter explanation because after-effects of intentional binding brought about by interval compression do not predict the illusory reversal of temporal order observed here. Our effects strongly suggest that the signals themselves are realigned rather than the perception of the interval between them. In the context of the current study, it remains unknown whether such recalibration involves a forward shift in time of sensory feedback or a backward shift of the motor action or, indeed, a combination of the two.

**Figure 5:**
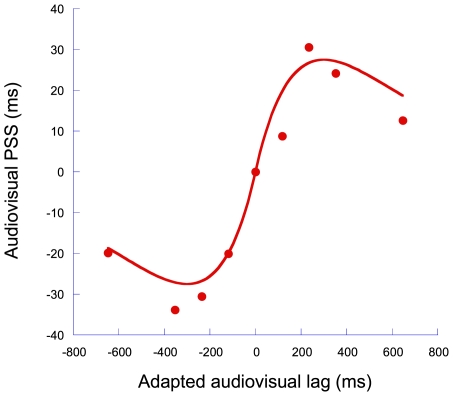
Adaptation to purely sensory asynchrony. Data taken from Fujisaki at al. (2004) where observers adapted to a fixed level of asynchrony between auditory and visual stimulus pairs before judging the relative temporal relationship of audiovisual test pairs [7]. PSS values are plotted as a function of the size of the adapting asynchrony and are expressed relative to the ‘adapt synchronous’ condition. The data are fitted with a same model used to fit the sensorimotor data shown in Figure 2 (see main text for details).

It seems logical to speculate whether the notion of an assumption of synchrony is limited to the perception of sensorimotor timing. Whilst an afferent self-generated sensory signal must be generated simultaneously with the completion of the causative motor action, audio-visual signals arising from a proximal external source - but independently of our motor actions - are also likely to share a correlated temporal register [47]. We applied the model described above to the audio-visual data of Fujisaki et al. [7], where audio-visual PSS was systematically mapped-out as a function of the magnitude of the adapting asynchrony. These data are shown in Figure 5. Clearly, Fujisaki et al's audiovisual effects are well described by this model (R^2^ = 0.943). Whilst the optimum exponent for this data set was similar to that found for our sensorimotor data (291±49 msec), a much smaller constant of proportionality is present (0.25±0.05). The smaller constant of proportionality perhaps suggests a weaker assumption of synchrony for sensory-sensory stimuli than for sensorimotor stimuli. Whilst the seen and heard components of audio-visual events will be generated simultaneously, they regularly arrive at their receptor surfaces with significant physical asynchrony (e.g. when observer-event distance is relatively large [5]). This provides an interesting avenue for future work. Audio-tactile and visuo-tactile signals are not subject to the significant environmental delays affecting their audiovisual counterparts. If long-term experience of this relationship drives a stronger assumption of synchrony between the signals, we would expect differences in the relationship between the extent of the recalibration and adapting asynchrony (e.g. Fig. 5) across the different multisensory pairings.

The nature of our adaptation effects is likely to have implications for their neural locus. The twin findings that (i) in terms of amplitude and tuning, all three sensorimotor pairings are recalibrated in a very similar manner *and* (ii) the illusion transfers to un-adapted sensorimotor pairings, strongly suggests that our effects are mediated at a relatively late-stage in the sensory and/or motor processing hierarchy. It seems reasonable to assume that the neural architecture subserving these effects is most likely located beyond the level of modality-specific brain areas [16].

In summary, our data suggest that temporal recalibration occurs because actions and their sensory consequences ‘should’ feel synchronous [48]. When this *a priori* assumption about the external world is combined with noisy sensorimotor estimates, adaptation initiates a realignment of our perception away from veridicality and toward the temporal relationship experienced during adaptation. Importantly, this only occurs when the nervous system can be confident that sensory inputs are a product of its own motor commands. Temporal discrepancies between motor actions and sensory events have been shown to be a powerful metric in the perception of causality [35], [36], [49] and the strength of this association declines exponentially with time [40]. This makes sense if the nervous system seeks to avoid potentially dangerous recalibration between our motor actions and sensory events with independent, external causes. An interesting direction for future work would be to use our paradigm to probe sensorimotor recalibration in schizophrenic patients with delusions of control. The work of two recent studies suggests that the temporal tuning of our effects (Fig. 2) may be considerably more narrow as a result of their tendency to attribute *external* causalities to *internally* generated stimuli [43], [45]. By the same token, it would be of interest to examine whether adaptive sensorimotor recalibration occurs when observers attempt to interpret actions and sensory consequences, but where the actions are generated by external agencies [24], [44].

## Materials and Methods

### Observers

Five trained observers (3 authors plus 2 naïve) participated in the within-modality experiments, whereas seven trained observers (3 authors, plus 4 naïve) participated in the crossmodal experiment. All experiments were run with the permission of The University of Bradford's ethics committee after gaining informed, written consent from all observers (in accordance with the Declaration of Helsinki).

### Stimuli

Visual stimulation was provided by a small (1.05° diameter) green LED (luminous intensity 600 cd/m^2^), auditory stimulation by a white noise burst (70 dB SPL), and tactile stimulation by a tap on the left forefinger delivered via an electrical solenoid. All stimuli were contained within 10 ms square wave-windowed temporal profiles. The relative timings of motor action and sensory stimuli were verified by simultaneous capture on a multiple trace oscilloscope. Auditory stimuli were delivered binaurally via tightly fitting, pinna-enclosing headphones (Sennheiser HD650). These headphones were worn by all observers for all experiments ensuring that the operational noise of the mousepress (see below) and solenoid remained inaudible to observers. The generation and presentation of all stimuli was controlled via custom-written software run in MatLab (Mathworks, U.S.A.) via a desktop PC.

### Procedures

During the experiment, observers were instructed to fixate the centre of the LED and press the mouse button at a pace of their own choosing. After each of the first four mousepresses, a stimulus was presented (‘flash’, ‘click’ or ‘tap’) at a constant delay of either 25, 50, 100, 200, 400 or 800 ms (‘adapting’ presentations). For all experiments this delay was consistent during a given experimental run. After the fifth mousepress, the same stimulus was presented with a delay of 25, 50, 75, 100, or 125 ms, which varied randomly within a method of constant stimuli (‘test’ presentation). Observers were required to judge whether the fifth stimulus presentation appeared *before* or *after* the fifth buttonpress, and make an unspeeded, binary forced-choice response via a computer keyboard. This response initiated the next cycle of adapting and test stimuli. Each of the five test delays was tested 10 times within an experimental run. Observers completed five runs for each of the three sensorimotor pairings tested, making a total of 750 test presentations per observer (10 presentations* 5 runs* 5 test delays * 3 sensorimotor pairings). The order of these runs was randomised so that each 10 repetition run was equally likely to contain any of the five different adapting delays.

The resultant psychometric functions (Fig. 1A) were fitted with a logistic function of the form
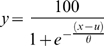
where µ is the sensorimotor asynchrony value corresponding to the PSS (the 50% response level on the psychometric function), and θ provides an estimate of temporal order threshold (approximately half the offset between the 27% and 73% response levels). In this way, PSS values were obtained for all observers in all of the conditions (Fig. 2).

The second, control experiment was identical to the audio-motor section of first experiment with the exception that observers changed their judgment type from ‘which came first, action or event?’ to ‘were action and event synchronous or asynchronous?’ The resultant data were fitted with Gaussian functions (Fig. 1B) of the form
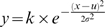
where µ is the sensorimotor asynchrony value corresponding to the PSS (the peak of the Gaussian function), σ provides an estimate of sensitivity to asynchrony (the width of the function) and k is a constant that reflects the amplitude of the function.

The final, crossmodal experiment was identical to the TOJ version of the initial within-modality experiment with two exceptions: (i) the adapting and test modalities differed, giving rise to six crossmodal conditions and, (ii) only the 200 ms adaptation delay condition was tested for each of these conditions (adapt V test A, adapt V test T, adapt A test V, adapt A test T, adapt T test V, and adapt T test A). The PSS and threshold values from these conditions are shown in Figures 3 and 4 respectively.
